# Three-dimensional soft tissue analysis of the face following micro-implant-supported maxillary skeletal expansion

**DOI:** 10.1186/s40510-018-0243-z

**Published:** 2018-11-19

**Authors:** Sara Abedini, Islam Elkenawy, Eric Kim, Won Moon

**Affiliations:** 1 Division of Growth and Development, Section of Orthodontics, School of Dentistry, Center for Health Science, University of California, Los Angeles, Room 63-082 CHS, 10833 Le Conte Avenue, Box 951668, CA, Los Angeles 90095-1668 USA; 2 Department of Computer Science, University of California, Los Angeles, 4732 Boelter Hall, Los Angeles, CA 90095 USA

**Keywords:** Skeletal expansion, Maxillary skeletal expander (MSE), Facial 3D images, 3D analysis, Miniscrews, Mini-implants, TAD anchorage, Rapid palatal expander

## Abstract

**Background:**

Skeletal maxillary expander (MSE) is one of the more recent expander designs being utilized for skeletal expansion by splitting the midpalatal sutures applying forces through palatal micro-implants. Its effects on the soft tissue remain a question asked by both patients and clinicians. The aim of this study was to analyze and quantify soft tissue facial changes induced by MSE.

**Methods:**

3D facial images (3dMD) were used to capture face scans of 25 patients generating 3D soft tissue meshes before expansion (T0), right after expansion (T1), and 1 year in retention (T2). MATLAB was then used, utilizing non-rigid iterative closest point algorithm, to align all samples in vertex correspondence and generate averages. Surface mapping of each average, along with its variance, allows for quantification of changes between the three pools of samples in 3D space.

**Results:**

The generated 3D p-maps between T0 and T1 demonstrate that statistically significant changes (*p* < 0.05 and *p* < 0.01) are localized in the circummaxillary area (paranasal, lips, and both cheeks). Vector map shows a mean displacement of 1.5 mm in the paranasal area. The right cheek showing a mean displacement magnitude of 2.5 mm while the left cheek has a mean of 2.9 mm. Direction of vectors are latero-anterior with more dominant anterior direction.

**Conclusions:**

There are significant changes in paranasal, upper lip, and at both cheeks following expansion using MSE with greater magnitude at the cheeks area. Those changes do not relapse after 1 year (*p* < 0.05).

## Background

Rapid maxillary expansion (RME) has been a common point of discussion and research for many years in Orthodontics field. Among the many kinds of expanders available, the majority have been studied and their effects have been documented. With the introduction of micro-implants, one of the most recent designs is the maxillary skeletal expander (MSE), which was developed for opening the midpalatal sutures and achieving a truly skeletal expansion [1–4]. Previous investigations on the effects of MSE on skeletal expansion and dentoalveolar bone bending in comparison with traditional RME or surgically assisted rapid palatal expansion (SARPE) have been conducted in two-dimensional (2D) or three-dimensional (3D) [5–7]. Many clinicians in Orthodontics or oral surgery have understood the importance of facial soft tissue to determine the limitations of treatment in order to have highly stable and functional treatment plan within the patient’s limitations of soft tissue adaptation and contours. However, the effects of expansion with MSE on soft tissue have remained poorly elucidated. Despite the great advancements in 3D technology, until recently scientists were relying on only 2D facial photographs to analyze the soft tissue changes. Lane and Harrell reported that the position of the head, distance between the camera and the subject, and the camera angle are all the factors that will result in unwanted discrepancies in the conventional method of photography [8]. These disparities provoke questions concerning the validity of quantitative information derived from this imaging system. On the other hand, landmark identification in the soft tissue is complicated due to the rounded and elastic nature of the tissue. Thus, in order to accurately evaluate the soft tissue, 3D imaging methods such as 3D computerized tomography (CT) and 3D facial scan images (3D-FSI) are needed [9, 10]. In a previous study, a new protocol for averaging multiple soft tissue surfaces and a new method to compare a single soft tissue surface map to the average was developed [11]. Utilizing the advanced technology of the 3dMD Face System (3dMD, Atlanta, GA), which is a very quick stereophotogrammetry system together with the newly developed method of 3D quantification, effects on the soft tissue following MSE expansion can be evaluated.

## Methods

### Study design

This study was approved by the institutional review board (IRB) of the University of California, Los Angeles. The study included 25 post-orthodontic treatment patients (9 males, 16 females) with a mean age of 21.3 years and the range spanned from 14.8 to 25.10 years of age. Three-dimensional facial soft tissue records using 3dMD of all patients diagnosed with transverse maxillary deficiency were taken before, right after expansion with MSE, and 1 year in retention. MSE design and expansion protocol were adopted as proposed by Cantarella et al. [11]. Patients selected were of various ethnicities, and inclusion criteria was the diagnosis of transverse maxillary deficiency based on the presence of either unilateral, or bilateral crossbite, or according to Andrew’s analysis of six elements [12]. Exclusion criteria were (1) all growing patients and (2) patients with craniofacial anomalies which could show changes in the soft tissues not related to expansion leading to false readings.

### Image acquisition, collection, and preparation

3dMD face system (3dMD, Atlanta, GA) combines structured light system technology with stereophotogrammetry. Furthermore, this system utilizes a synchronized multi-camera configuration with three cameras on each side (one color, two infrared) that can capture high-resolution images in ~ 1.5 ms [8]. A natural and clinically reproducible head position was adopted for all the patients. Images were taken in the natural head position (NHP) for every selected patient at three time points. The first time point (T0) is taken right before MSE placement. The second time point (T1) was taken once expansion is complete. The third time point (T2) is the retention time point, which would be taken 1 year following termination of expansion.

To maintain a consistent standardized protocol for image capturing and to ensure reproducibility, multiple picture for each patient were taken in the NHP and at different time points. 3dMD system cameras were calibrated according to manufacturer instructions before every patient’s scan is captured. All images were taken at repose state, with lips relaxed in a resting position. Since the captured images are rendered within less than a minute, the protocol for 3dMD image capturing requires that the rendered meshes are checked immediately for any artifacts or incorrect patient/lip position. OBJ files of all images generated by 3dMD for all time points are then loaded into MATLAB v9.1 (The MathWorks, Inc., Natick, MA, USA) for processing in the following steps.

#### Global preliminary alignment and mesh preparation

**Fig. 1:**
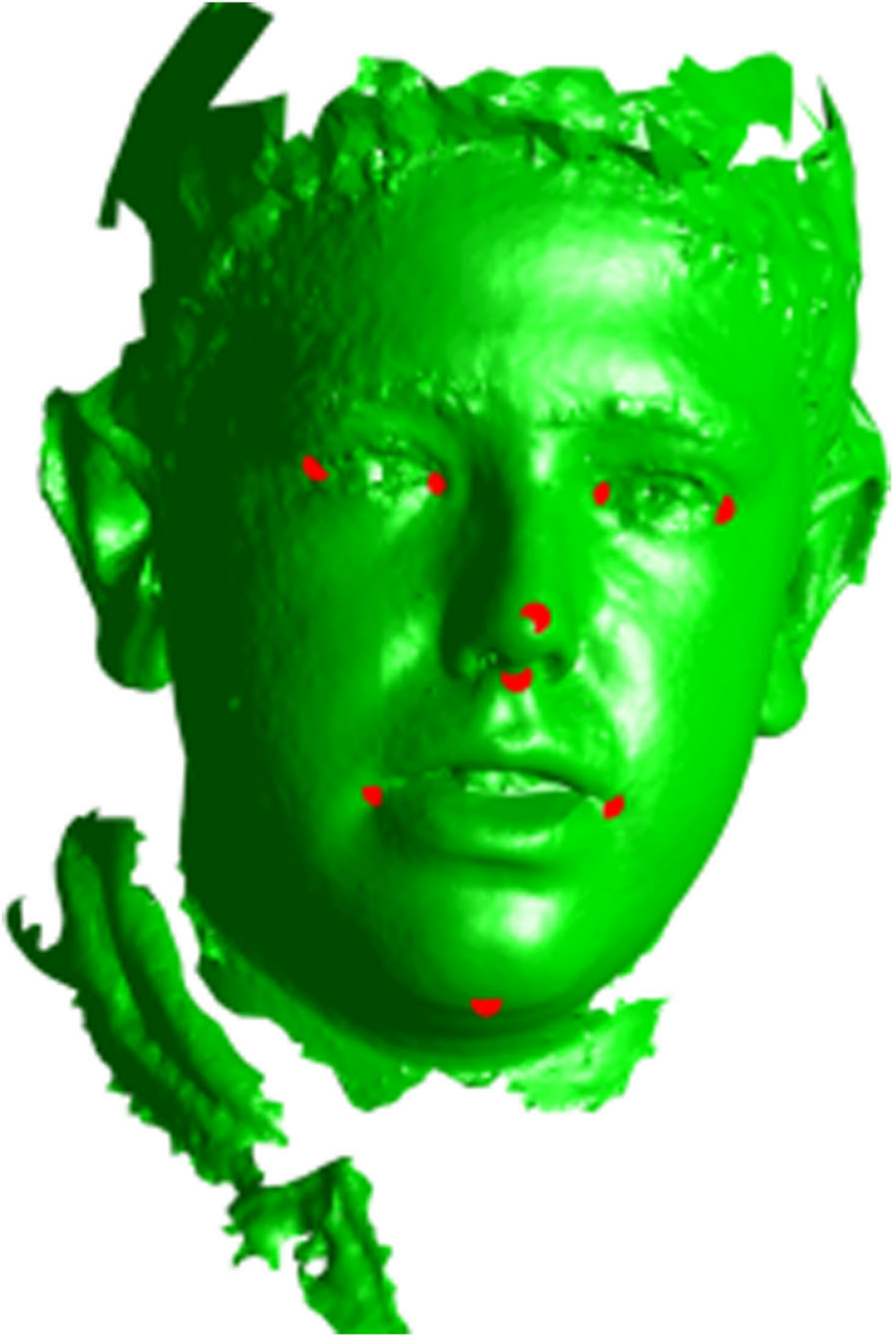
Nine facial landmarks used for global alignment (left eye outer, left eye inner, right eye outer, right eye inner, nose tip, nose base middle, mouth left corner, mouth right corner, chin middle)

**Fig. 2:**
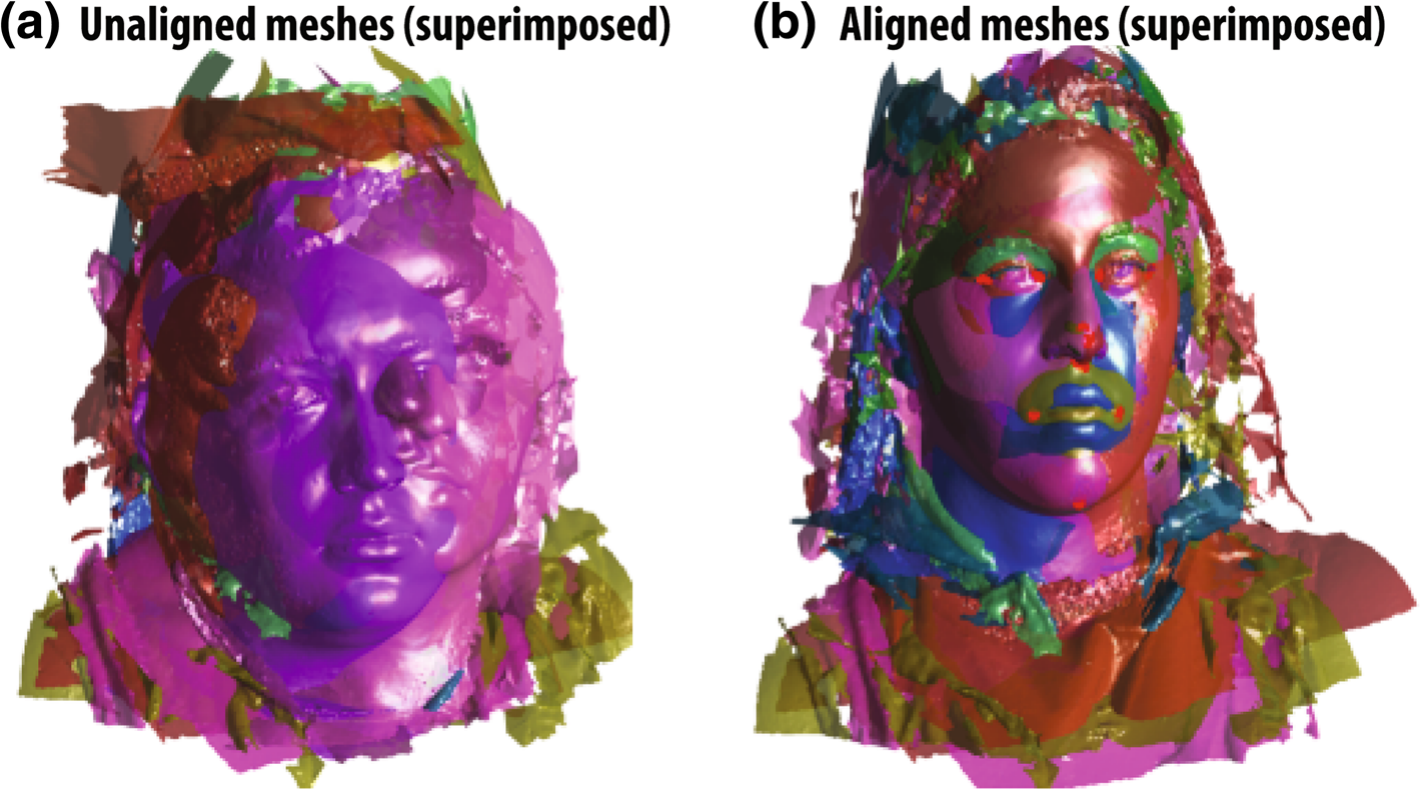
Applying generalized Procrustes analysis (GPA) transforms to meshes. **a** Before meshes are aligned. **b** After aligning the meshes by translation, rotation, and scaling using nine facial landmarks

**Fig. 3:**
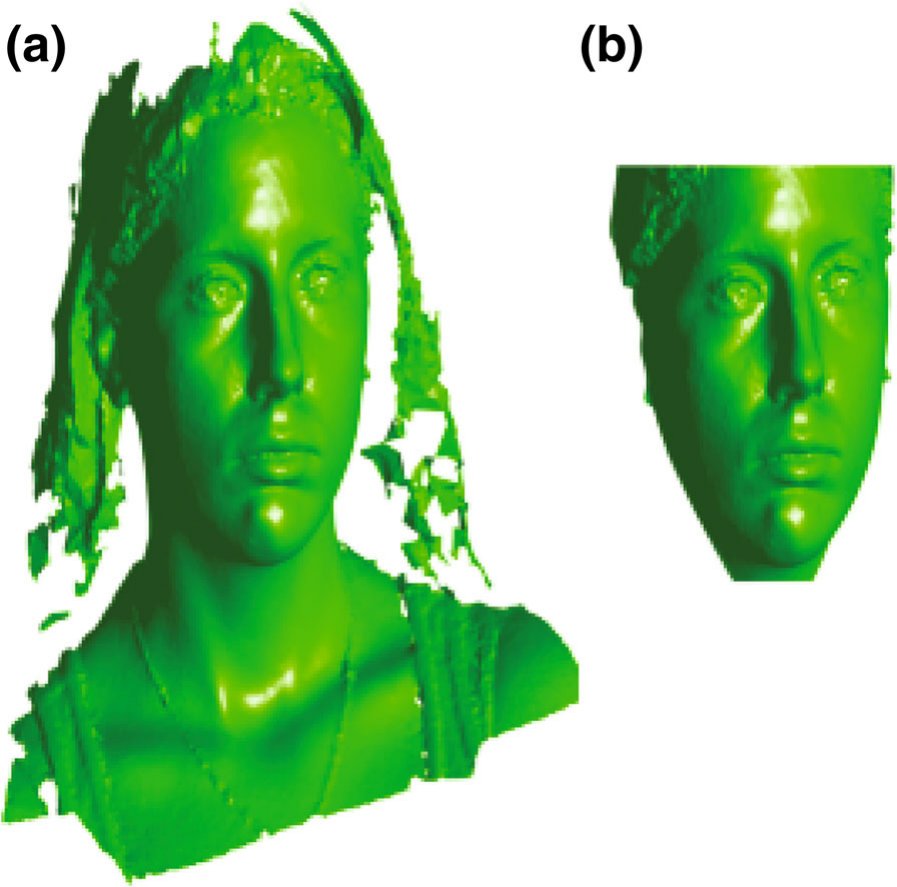
Mesh clipping with Slicer software, a sample mesh before clipping (**a**) and after clipping all excess structures (**b**)

**Fig. 4:**
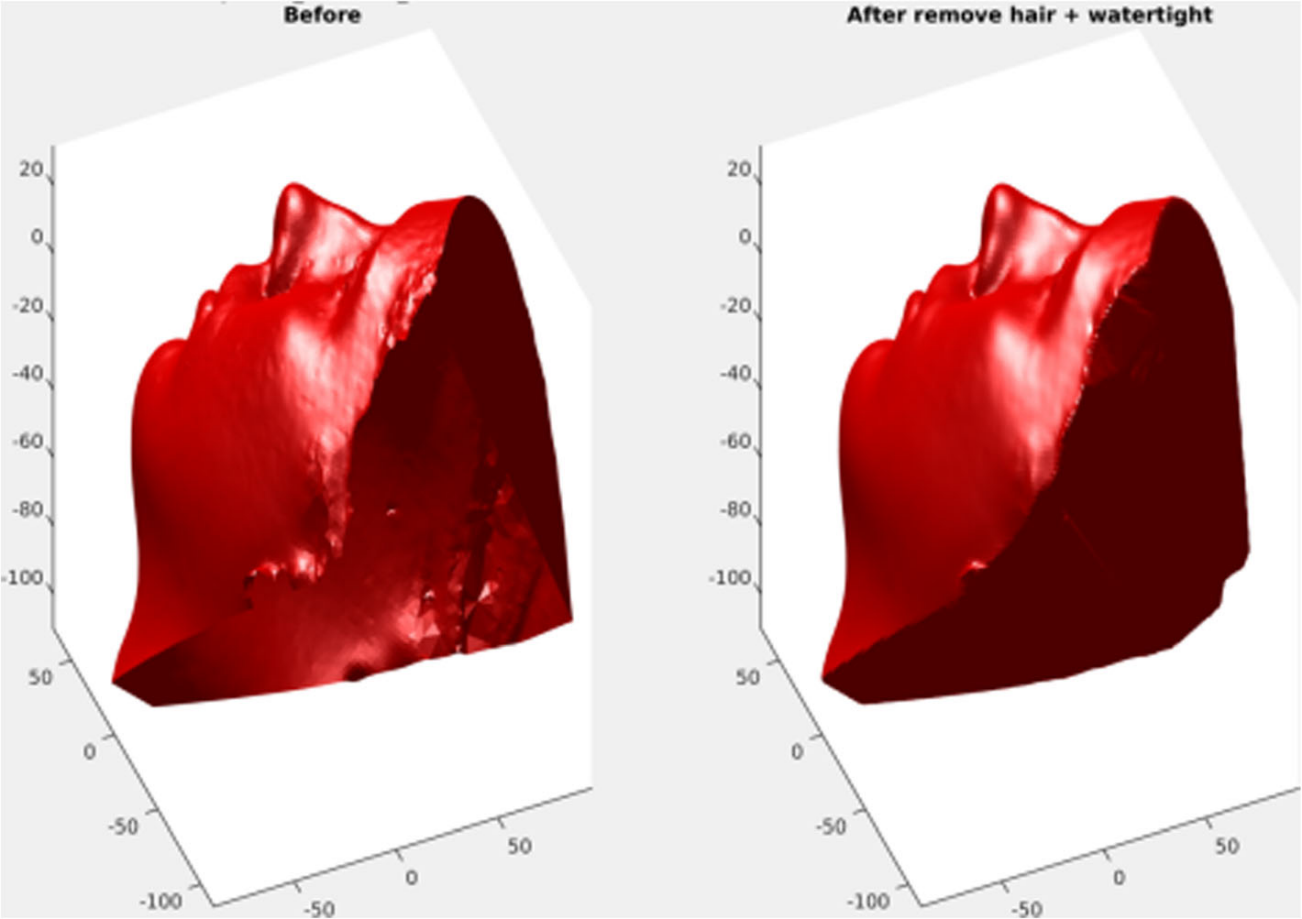
The meshes are made watertight to facilitate the computation of the soft tissue analysis. A sample mesh before (left) and after (right) closing off the meshes from the posterior side

To initially align the samples, nine manually labeled facial landmarks (left eye outer, left eye inner, right eye outer, right eye inner, nose tip, nose base middle, mouth left corner, mouth right corner, chin middle) were applied on each sample (Fig. 1). These landmarks were only used for initial rough alignment and its purpose is only to decrease the computing resources needed for the actual alignment utilizing each vertex, therefore any inaccuracy was eliminated later on. Global similarity transformation matrix (translation, rotation, scaling) was computed to align all meshes together using the previously labeled landmarks. The computed transformations were done utilizing generalized Procrustes analysis (PGA) on MATLAB [13] (Fig. 2). All of the aligned meshes are then imported into 3DSlicer 4.5.0 open-source software (http://www.slicer.org) in their original .OBJ format [14]. Autodesk MeshMixer (Autodesk, Inc., CA, USA) is then utilized to manually clip all the excess irrelevant structures in the mesh (neck, body, long hair, clothing) without compromising any of the facial structures (Fig. 3), and the meshes were closed from the posterior side to make them watertight which is crucial for the final correspondence steps (Fig. 4).

#### Establishing vertex correspondence

In order to accurately align the different meshes, correspondence has to be established between each vertex on every face. For example, every vertex on PatientFace1 has to be in direct correspondence with every vertex on PatientFace2, PatientFace3, etc.

**Fig. 5:**
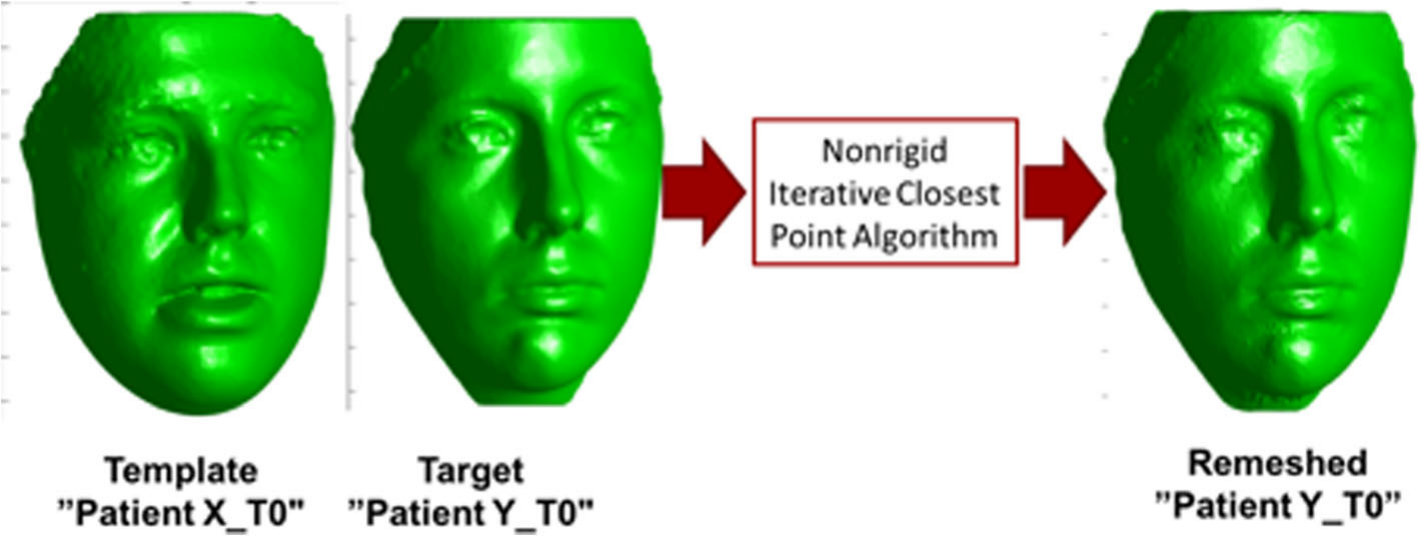
Illustration of how to establish vertex correspondence between meshes. Template mesh is being remeshed (deformed) to match the target face (mesh) using non-rigid ICP. Remeshed face (on the right) is now representing the target mesh and it will be in vertex correspondence with the original template

The task of establishing vertex-level correspondences between two 3D meshes has been an active area of research in computer graphics and computer vision for many years [15]. One way of doing this is by a process of remeshing (deforming) a single face (template) to match every other face (target) from the whole sample set. For example, Patient1_T0 would be used as a template, it will be remeshed to match every other patient (Patient2_T0, Patietn3_T0, etc.). The remeshed (deformed) template would represent the target mesh accurately, and it will be in vertex correspondence with the original template, thereby achieving our goal and by repeating this process on every other face, we would establish vertex correspondence between the entire set of samples. This process of remeshing was done using non-rigid iterative closest point algorithm (non-rigid ICP) on MATLAB v9. [16] (Fig. 5).

### Computing average meshes

Once vertex correspondence was established between all the samples, the next step was to average all aligned meshes. This stage was done by computing the average position of each vertex between all T0, T1, and T2 samples, generating three averages (T0_Average, T1_Average, and T2_Average). Average meshes represent a collection of each vertex on all the samples averaged, with the individual values representing the variance. Both the average and the individual vertex values were used to compute the statistical significance.

### Comparison and quantification

For comparison, a standard *T* test could only give us three different *p* values one for each axis. To defeat this limitation, we opted to use a statistical function called “Multivariate Paired Hotelling’s T-Square”; this function allows us to input our values as a matrix taking into consideration the exact coordinate of each vertex on all three axes at the same time, thereby factoring in the variance automatically [17]. This test was used to compare pre-expansion vertex parameters to post-expansion parameters, taking into consideration the sample mean vectors, and the variance of each vertex. MATLAB was used to compute the *p* value generated using Hotelling’s T-Square test, and due to the number of vertices involved (around 30,000), p-maps was utilized in lieu of simple *p* values, between T0 and T1, T1 and T2, and T0 and T2. Two p-maps were generated for each time point; one showing areas of statistical significance (*p* < 0.05), and one showing areas of high statistical significance (*p* < 0.01).

### Splitting into right and left groups

In a recently published article on the same expander design, data showed noticeable asymmetry in expansion patterns with MSE [11]. Therefore, to eliminate any discrepancy in our results that may occur due to averaging out all the samples, the samples were divided into right and left expanded groups for an additional group of statistical tests.

Establishing whether a specific sample was expanded more to the right or left, or was equally expanded, cone beam computed tomography (CBCT) of the patients were utilized. All patients diagnosed with transverse maxillary deficiency (TMD) at this institution undergo CBCT scans before and after expansion as part of their protocol; therefore, no additional X-ray exposure was needed. Asymmetry was evaluated on the CBCT’s using the method used by Cantarella et al. [11]. P-maps were then created for each of the two new groups (*n* = 11 in each group). Samples of equal expansion (*n* = 4) were left out of both of these groups and were evaluated only in the original sample pool of *n* = 25.

## Results

**Fig. 6:**
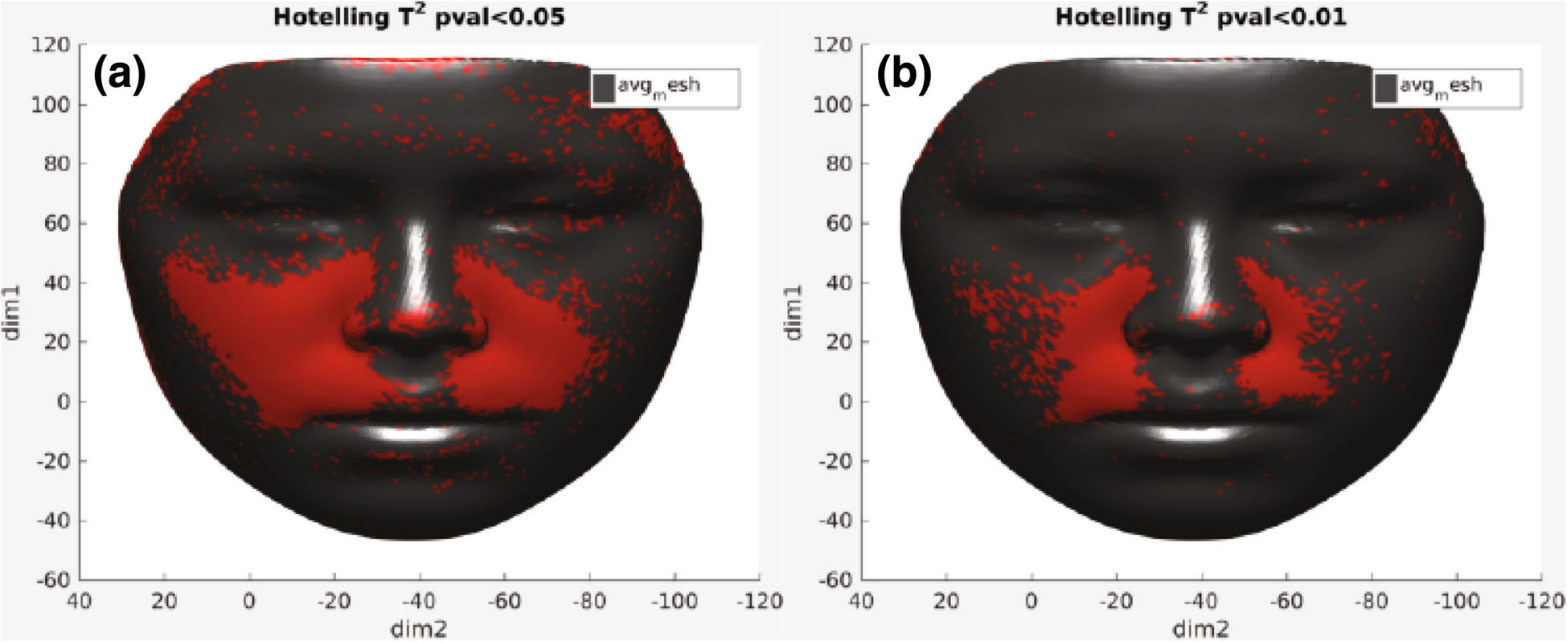
P-map illustration for comparison of T0 (initial, right before MSE placement)–T1 (immediately after expansion) time points with *p* < 0.05 (**a**) and *p* < 0.01 (**b**)

**Fig. 7:**
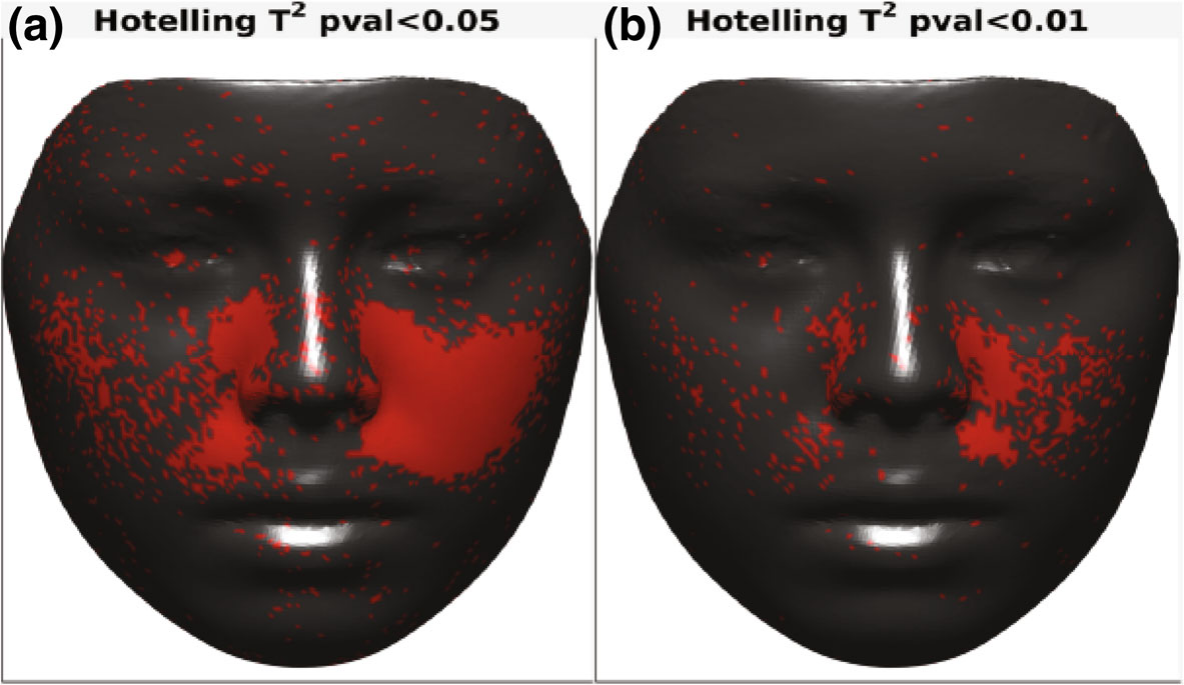
P-map illustration for comparison of T0 (initial, right before MSE placement)–T2 (retention, 1 year after termination of expansion) time points with *p* < 0.05 (**a**) and *p* < 0.01 (**b**)

**Fig. 8:**
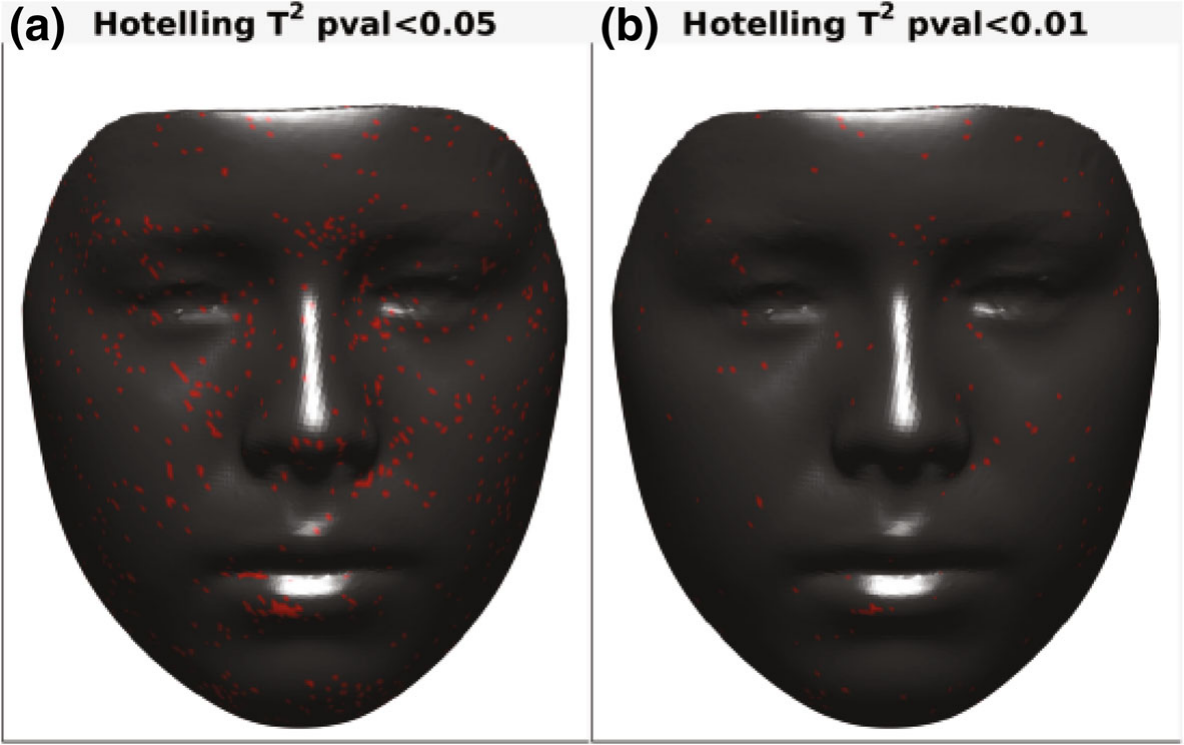
P-map illustration for comparison of T1 (immediately after expansion)–T2 (retention, 1 year after termination of expansion) time points with *p* < 0.05 (**a**) and *p* < 0.01 (**b**)

**Fig. 9:**
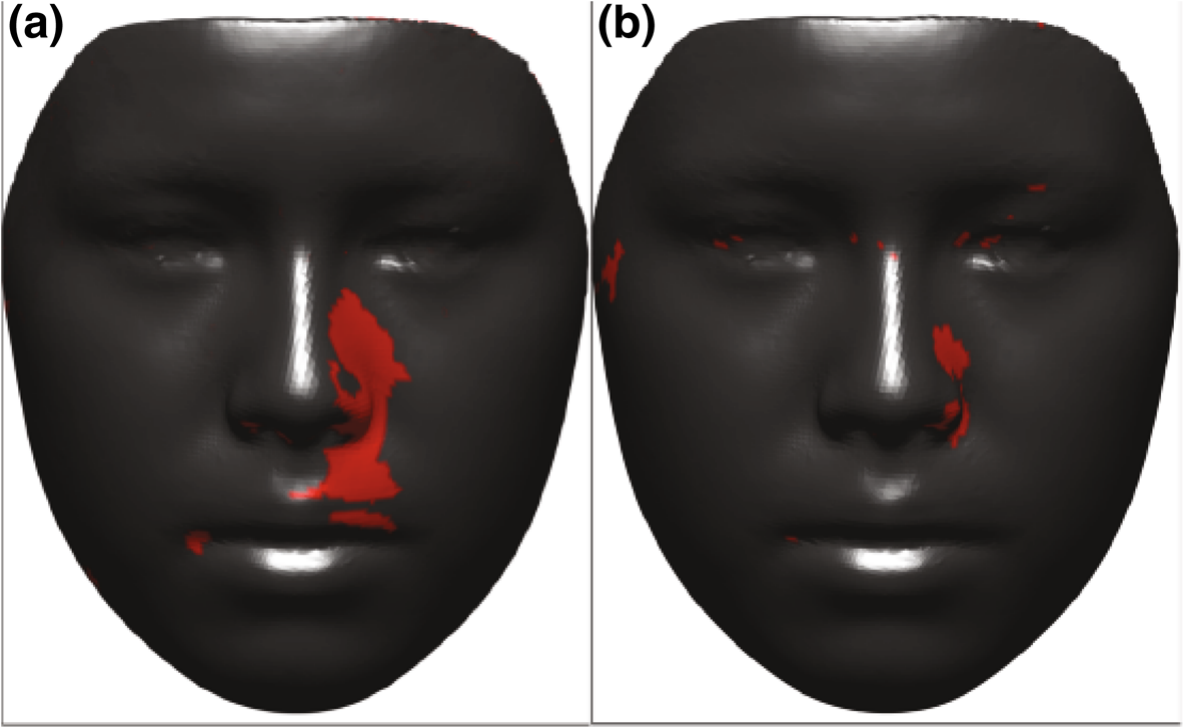
P-maps between T0 (initial, right before MSE placement) and T1 (immediately after expansion) time points for left side expanded group only with *p* < 0.05 (**a**) and *p* < 0.01 (**b**)

**Fig. 10:**
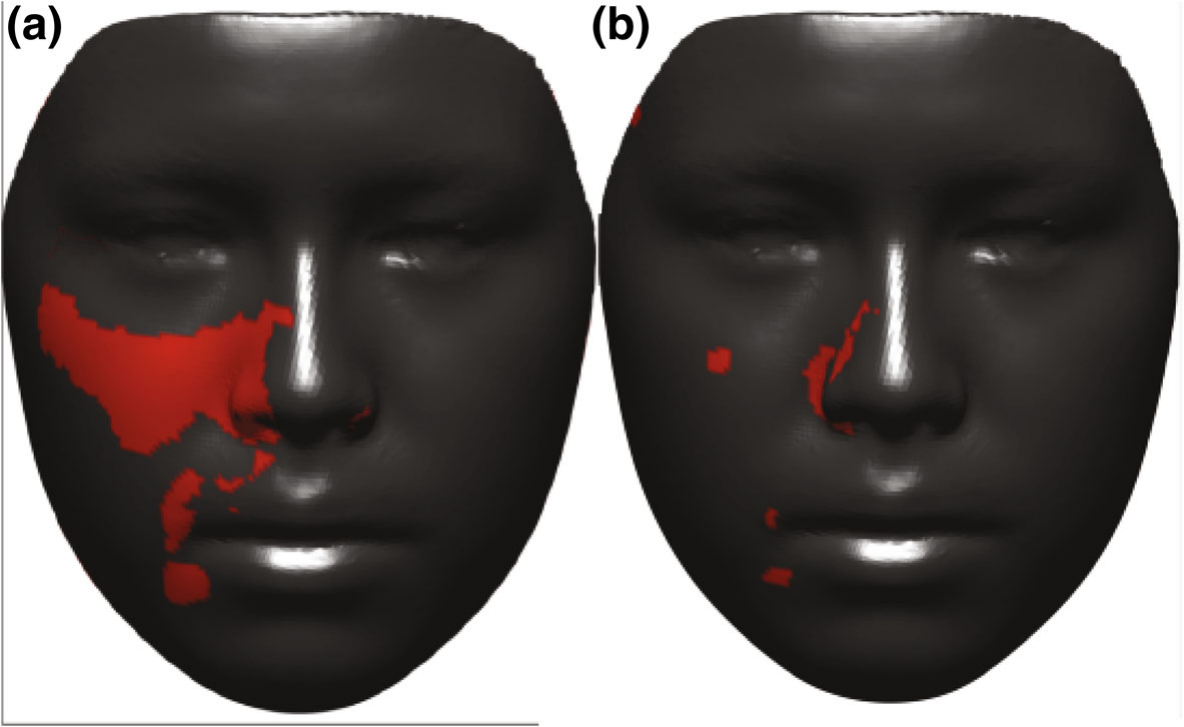
P-maps between T0 (initial, right before MSE placement) x and T1 (immediately after expansion) time points for right side expanded group only with *p* < 0.05 (**a**) and *p* < 0.01 (**b**)

To evaluate the effects of expansion on facial soft tissue, p-maps were created between each two time points (T0–T1, T0–T2, and T1–T2) and with two *p* values of *p* < 0.05 and *p* < 0.01 to facilitate illustration of changes in the face. Figure 6 presents p-maps between T0 (initial right before MSE placement) and T1 (immediately after expansion) using the co-ordinates of each vertex as the parameter on all time points with *p* < 0.05 (a) and *p* < 0.01 (b). The red areas denoted in the (a) figure are considered areas of statistically significant (around the nose (paranasal), upper lip, cheeks, and to some extent zygomatic arch); however, highly statistically significant areas appear to be concentrated more around inner cheek, paranasal, and upper lip (b). Figure 7 shows p-maps between T0 and T2 (retention, 1 year after termination of expansion) time points with *p* < 0.05 (a) and *p* < 0.01 (b). P-map in this figure is nearly following the same pattern as Fig. 6, which is appearance of significant changes around the paransal areas and inner cheek but with less concentration. Figure 8 illustrates p-maps between T1 and T2 time points with *p* < 0.05 (a) and *p* < 0.01 (b) to evaluate any possible relapse in soft tissue changes after one-year retention. Scattered red areas indicate that there were very minimal to none soft tissue changes during the 1-year retention period. Figures 9 and 10 illustrate the p-maps between T0 and T1 time points but using only the samples which have dominantly expanded toward the left and right side, respectively. The left side dominant samples showed significant changes only on the nose and cheek of the left side and there were no significant changes on the right side (Fig. 9). Same results were observed for the right side expanded samples with no significant changes occurred on the left side (Fig. 10).

**Fig. 11:**
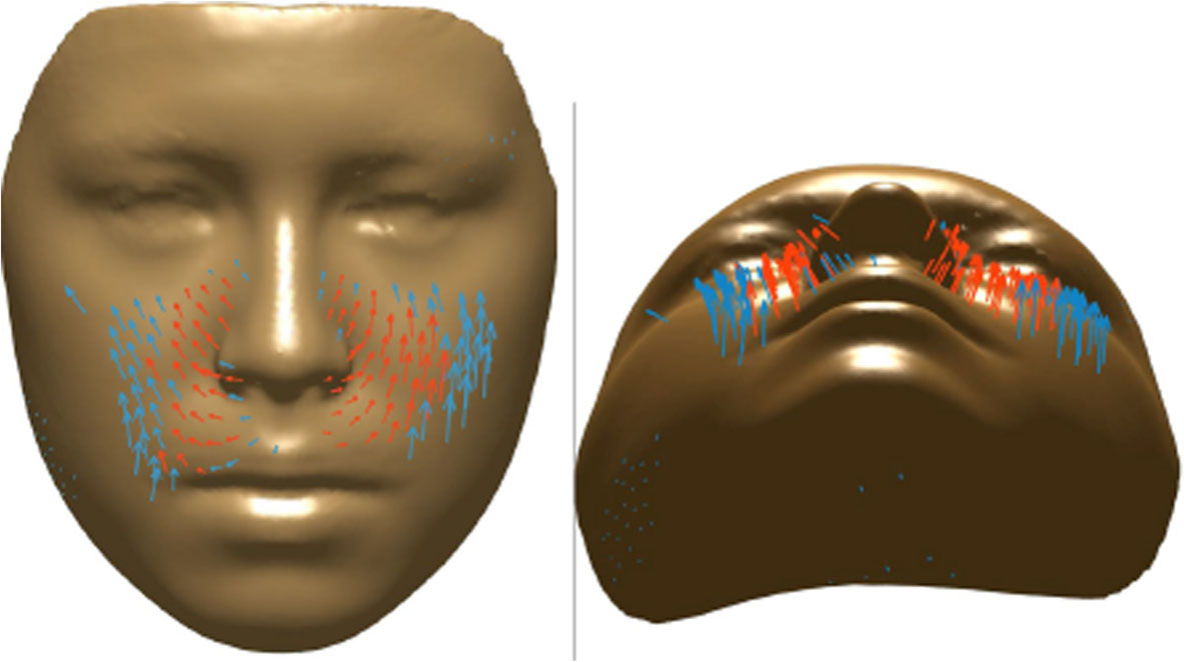
Vector map presents the direction of changes between T0 (initial, right before MSE placement) and T1 (immediately after expansion) time points. Blue arrows denote vectors with *p* < 0.05, and the red ones denote vectors with *p* < 0.01

For further analysis of the statistically significant changes, vector maps showing direction of changes have been made. Figure 11 shows a vector map between T0 and T1 where the red arrows denote vectors with *p* < 0.01, and the blue ones denote vectors with *p* < 0.05. The vectors shown are a condensed representation where each vector represents an average of all the vectors in a five-unit neighborhood, so as to better visualize the vectors’ direction. As it is shown in those figures, direction of the vectors is toward lateral and anterior of the face with more dominant forward direction.

**Fig. 12:**
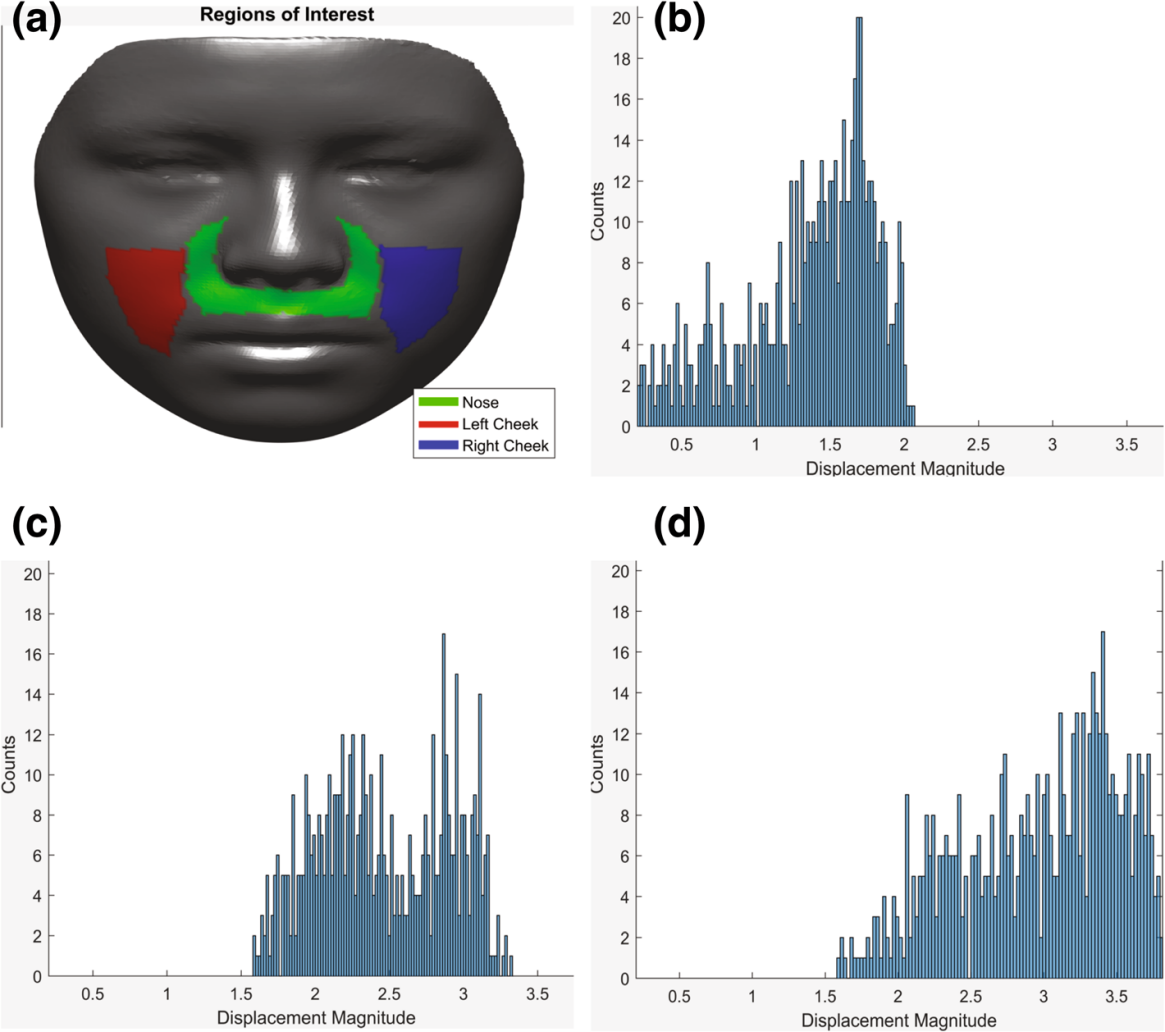
Face divided in three main areas of nose, right and left cheek to quantify the vectors (**a**). Magnitude of the vectors representing changes in each area presented in histogram (**b**–**d**). *X*-axis represents the vectors’ magnitude and *Y*-axis represents the number of vectors of each variable magnitudes

For more in-depth statistics, the three major regions of significance (nose (refer to paranasal and upper lip regions together), right cheek, and left cheek) were chosen to quantify the vectors (Fig. 12a). Figure 12b–d illustrates the vectors’ magnitude in histogram format based on each region. In each histogram, *Y*-axis represents the number of vectors of variable magnitudes and *X*-axis represents the vectors’ magnitude, showing the distribution within each area.

Table 1 presents the breakdown of the data within each histogram, showing minimum/maximum displacements, means, medians, and standard deviations of displacement vectors for each region. Based on the means, the displacement magnitude appears to be larger in both right and left cheeks compared to the nose area (paranasal and upper lip).

**Table 1:** Breakdown data of the vectors’ magnitude in all three major areas of the face presented in Fig. 12a

	Min	Max	Mean	Std	Median
Nose	0.2023	2.063	1.3618	0.4473	1.4671
Right cheek	1.5885	3.3252	2.4614	0.4399	2.422
Left cheek	1.5898	3.8134	2.9572	0.5462	3.0529

## Discussion

Utilizing 3D facial scans in orthodontics is relatively new, and although it presents an unprecedented solution to the inaccuracy of using photography to assess soft tissue changes, there are still many obstacles to achieving true 3D quantification of soft tissues. The averaging of a 3dMD face was previously accomplished in an earlier study, establishing a method by which multiple 3dMD scans can be aligned and averaged into one [18]; this concept was modified and applied to clinical orthodontics in this project.

Our goal of achieving vertex-to-vertex correspondence was achieved by the non-rigid iterative closest point algorithm [16], which is now easily reproducible through MATLAB; this algorithm has been validated for its ability to deform (reparametrize) one mesh (template) to match all the other sample meshes (targets), whereas this deformed mesh will represent each target mesh after achieving vertex-to-vertex correspondence with the template. Rigid registration works by yielding a transformation that does not change the distance between any two points [19]. For instance, the entire face could be rotated, translated, or scaled as a whole, but one area of the mesh cannot move independently of other areas; this makes rigid registration a very poor choice for quantifying soft tissue changes which are almost always localized. On the other hand, non-rigid registration usually deals with affine transformations such as scaling and shear mapping and it is usually nonlinear transformations where a localized area of the mesh can translate, leading to a change in the distance between vertices [20]. This critical difference deemed non-rigid registration as the method of choice in our study assessing the soft tissues.

The effect of expanders on the soft tissues has never been studied and that could be due to the assumption that it is expected to be minute, or overshadowed by other growth changes and therefore hard to evaluate. However, with the introduction of MSE, more non-growing patients can now be expanded skeletally, which called for a more in-depth study on the actual effects of expansion on the soft tissues, utilizing the previously mentioned methodology. The results showed expected areas of change to be statistically significant (*p* < 0.05), while some specific areas showed highly significant (*p* < 0.01) changes after expansion with MSE. These figures were reached after applying the Hotelling’s multivariate test, a statistical test that can be accurately utilized when dealing in 3D space. This test was able to consider movements in all three axes simultaneously; for example, if a particular vertex was considered statistically insignificant in *Y*- and *Z*-axes, but significant in *X*-axis, it may still be statistically significant overall, depending on the variance which is factored in the matrix input of the Hotelling’s test.

Overall, the areas of most change appear to be around the nose (paranasal area) and on the medial sides of both cheeks, but as previously mentioned, the nature of expansion appears to be asymmetric, usually yielding more soft tissue changes on one side than the other. Our samples in the end appear to give symmetric changes which is simply due to the fact that almost half of the samples were dominantly expanded to the right side (*n* = 11) while the other half were expanded more toward the left side (*n* = 11). The separated p-maps clearly show this nature of asymmetry as when the right-sided samples are grouped together, the entire left side is statistically insignificant, and vice versa.

Vector maps were then created to demonstrate the magnitude and direction of such change. In the paranasal area, the vectors appear to be moving forward and outward; however, the horizontal forward component seems to be greater which validates the proposed pattern of expansion from a previous study by Cantarella et al. [11]. The vectors in the cheek areas show lateral and forward movement with higher magnitude. The mean magnitude was 2.4 mm on the right cheek and 2.9 mm on the left cheek, while the mean magnitude in the nose (paranasal and upper lip) area was 1.3 mm (Table 1).

This method was able to quantify the changes in 3D soft tissue between two groups in a reproducible manner; the effects of MSE expansion were assessed and visualized. In this method, we could overcome the need of radiation exposure (CBCT) to evaluate the soft tissue changes as was previously the only available resources to perform 3D studies [21]. We can now predict the effect of MSE on the face to an extent. It is of utmost importance to consider other factors that can have effect on the soft tissue changes over time. Such as growth and weight changes. However, in this particular study, confounding factors appear to have been minimized due to the following. First of all, patients that are going under MSE expansion are generally patients that have passed their growth spurt and therefore use of regular expanders such as Haas Hyrax are not indicated. We have eliminated the patients with potential growth from the sample size. Secondly, following standard expansion protocol with MSE [11], full expansion is achieved rapidly resulting in a short time period between T0 and T1 interval minimizing the amount of possible change that is due to growth, rather than expansion. Finally, the soft tissue results appeared to coincide with previously published results assessing the skeletal changes following MSE expansion using CBCT images [11]. Further studies utilizing correlation analysis are needed to confirm and validate this assumption.

However, predicting the nature of asymmetry and whether it would be equal expansion or dominantly expanding toward one side of the face remains unclear that will be the subject of a future study to follow. In addition, other topics such as correlating the amount of soft tissue changes with the amount of skeletal expansion or longer retention follow ups need to be elaborated more in future studies.

The developed method can be utilized to quantify many other treatment modalities with high impact on clinical application eventually helping the clinician be able to assess consequences of treatment on the face. With continuing advances in orthodontics, this can soon be applied to predict any treatment modalities effect on a patient’s scanned 3D face, which would be the ultimate goal of our study.

## Conclusions

- The developed protocol can be used to assess and quantify soft tissue changes due to different treatment modalities.
- Expansion with MSE has statistically significant impact on the soft tissue of the face, in particular paranasal and cheeks area. Changes are stable after 1-year retention.
- The mean magnitude of change is higher around the cheeks compared to the paranasal area.
- The soft tissue expands in a forward and lateral direction, primarily in the forward direction.

## Abbreviations

CBCT: Cone beam computed tomography
MSE: Maxillary skeletal expander
NHP: Natural head position
RME: Rapid maxillary expansion
SARPE: Surgically assisted rapid palatal expansion
TMD: Transverse maxillary deficiency
